# Genetic control of compound leaf development in the mungbean (*Vigna radiata* L.)

**DOI:** 10.1038/s41438-018-0088-0

**Published:** 2019-02-01

**Authors:** Keyuan Jiao, Xin Li, Shihao Su, Wuxiu Guo, Yafang Guo, Yining Guan, Zhubing Hu, Zhenguo Shen, Da Luo

**Affiliations:** 1 Institute of Traditional Chinese Medicine and Natural Products, College of Pharmacy, Jinan University, Guangzhou, China; 2 College of Life Sciences, Laboratory Center of Life Sciences, Nanjing Agricultural University, Nanjing, China; 3 Guangdong Key Laboratory of Plant Resources, School of Life Sciences, Sun Yat-Sen University, Guangzhou, China; 4 Institute of Plant Stress Biology, State Key Laboratory of Cotton Biology, Department of Biology, Henan University, Kaifeng, China

**Keywords:** Leaf development, Plant development

## Abstract

Many studies suggest that there are distinct regulatory processes controlling compound leaf development in different clades of legumes. Loss of function of the *LEAFY* (*LFY*) orthologs results in a reduction of leaf complexity to different degrees in inverted repeat-lacking clade (IRLC) and non-IRLC species. To further understand the role of *LFY* orthologs and the molecular mechanism in compound leaf development in non-IRLC plants, we studied leaf development in *unifoliate leaf* (*un*) mutant, a classical mutant of mungbean (*Vigna radiata* L.), which showed a complete conversion of compound leaves into simple leaves. Our analysis revealed that *UN* encoded the mungbean LFY ortholog (VrLFY) and played a significant role in leaf development. In situ RNA hybridization results showed that *STM*-like *KNOXI* genes were expressed in compound leaf primordia in mungbean. Furthermore, increased leaflet number in *heptafoliate leaflets1* (*hel1*) mutants was demonstrated to depend on the function of *VrLFY* and *KNOXI* genes in mungbean. Our results suggested that *HEL1* is a key factor coordinating distinct processes in the control of compound leaf development in mungbean and its related non-IRLC legumes.

## Genetics: Leaf architecture gene evolved early in legume plants

A legume gene involved in determining the spatial pattern of leaflets seems to have evolved this function earlier than previously thought. Xin Li from Nanjing Agricultural University, China, and coworkers studied a mutant variety of mungbean in which the plant’s leaves display a simple architecture instead of the usual compound arrangement, with several leaflets joined to a single stem. They discovered a mutation in the mungbean version of a gene called *LEAFY*. This gene, together with others, proved pivotal in compound leaf development in mungbeans. The results run counter to prior data showing that, outside of a small group of related legume species, *LEAFY* plays only to play a minor role in compound leaf development in these agriculturally important plants. The findings could thus inform breeding efforts in legumes generally.

## Introduction

Plant leaves are the primary photosynthetic organs that are initiated on the flanks of the shoot apical meristem (SAM). The class I *KNOTTED1*-like homeobox (*KNOXI*) genes are involved in the maintenance of the meristem activity of SAM, while the initiation of leaves requires downregulation of *KNOXI* genes at the incipient site^1–3^. In simple-leafed species such as *Arabidopsis thaliana*, downregulation of *KNOXI* genes in leaf primordia is permanent, whereas in most compound-leafed eudicot species, including the tomato (*Solanum lycopersicum*) and *Cardamine hirsuta*, *KNOXI* genes are reactivated in leaf primordia after initiation of leaf development^4–6^. In *C. hirsuta*, the leaflet number is reduced in mutants of the *KNOXI* gene *SHOOTMERISTEMLESS* (*ChSTM*) or *BREVIPEDICELLUS* (*ChBP*)^7,8^. In *S. lycopersicum*, ectopic expression of the *KNOXI* genes *Tomato KNOTTED1* (*Tkn1*) or *Tkn2* (orthologs of *STM* and *BP* in tomato, respectively) in transgenic lines, or upregulated expression of *Tkn1* or *Tkn2* in related mutants, results in ramification for compound leaves suggesting that regulatory processes mediated by *KNOXI* genes, especially *STM/BP*-like *KNOXI* genes, play pivotal roles in compound leaf development^5,9,10^.

However, in the inverted repeat-lacking clade (IRLC) of legumes, which includes *Pisum sativum* and *Medicago truncatula*, the expression of *STM/BP*-like *KNOXI* genes is excluded from leaf primordia^11–13^. Genetic analysis shows that single mutants, double mutants and triple mutants of 3 *STM/BP*-like *KNOXI* genes, namely, *MtKNOX1*, *MtKNOX2*, and *MtKNOX6*, in *M. truncatula* do not show obvious defects in compound leaves^13^. Thus, *STM/BP*-like *KNOXI* genes may not be involved in compound leaf development in IRLC legume plants^11–13^. Instead, another type of transcription factor, UNIFOLIATA (UNI) in *P. sativum* and SINGLE LEAFLET1 (SGL1) in *M. truncatula*, orthologs of LEAFY (LFY) from *Arabidopsis*, functions in controlling compound leaf development^14–16^. The *uni* mutants in pea and *sgl1* mutants in *M. truncatula* exhibit single leaflet phenotypes, and inflorescence and floral defects^15,16^. Hence, the LFY orthologs appear to play a significant role in compound leaf development in IRLC legumes. Furthermore, it has been shown that the UNI cofactor UNUSUAL FLORAL ORGANS (UFO) in pea, and PALM1, an upstream transcription factor of *SGL1* in *M. truncatula*, are involved in the control of leaf complexity^17,18^. Recent studies show that the adaxial–abaxial regulators PHANTASTICA (PHAN), ARGONAUTE7 (AGO7), and AUXIN RESPONSIVE FACTOR 3 (ARF3) regulate the expression level of *PALM1* and therefore control compound leaf development in *M. truncatula*^19–21^.

The function of the *LFY* orthologs during compound leaf development has also been investigated in non-IRLC legumes, including soybean and *L. japonicus* in which KNOXI proteins are expressed in leaves, and are likely associated with compound leaf development^12,22^. In *L. japonicus*, a mutant of the *LFY* ortholog *Proliferating Floral Meristem* (*PFM*) exhibits one or two reduced basal leaflets^12^. In soybean *LFY*-RNAi-silenced lines, only the leaflet number of the compound leaves produced at the second node is reduced^12^. This would indicate that there is a minor role in compound leaf development for *LFY* orthologs in non-IRLC legume species^12,22,23^.

In this study, we described the compound leaf developmental processes in a non-IRLC legume species, mungbean (*Vigna radiata* L.), a fast-growing (60–90 days) warm-season grain legume, and characterized the *unifoliate leaf* (*un*) mutants that showed a complete conversion of compound leaves into simple leaves. Four alleles of *un* carried mutations in the *LFY* ortholog, indicating that the *LFY* ortholog in mungbean played a significant, rather than a minor role in compound leaf development. Phylogenetic analysis of the KNOX gene family in legumes was conducted, and the expression of four *STM/BP*-like *KNOXI* genes was characterized in mungbean using in situ RNA hybridization. Furthermore, genetic interaction and gene expression analysis showed that increased leaflet number in *heptafoliate leaflets1* (*hel1*) mutants involved regulatory processes mediated by *VrLFY* and *STM/BP*-like *KNOXI* genes in mungbean. This study showed that the LFY ortholog might play a more significant role in the control of compound leaf development earlier than the time estimated by Champagne et al.^12^.

## Materials and methods

### Plant material and growth conditions

All the mutants were isolated from the M_2_ population of a mutagenized mungbean cultivar, Sulu, generated in Nanjing, China. The gamma irradiator was calibrated to irradiate the seed lots with 400 Gy of gamma rays. The M_1_ seeds were sown in the field, and the M_2_ seeds were individually harvested from the population. Approximately 36 seeds of each M_2_ line were planted in individual rows in the field, with a distance of 0.3 m between rows. The mutant plants were then individually harvested and sown for further observation in the greenhouse at 26–30 °C with a 16-h light/8-h dark photoperiod at 200 μmol m^−2^ s^−1^. The allelic nature of genes was confirmed by crosses among *un1-1*, *un1-2*, *un1-3*, and *un1-4*, using heterozygote parents because the mutants were sterile (the mutant plants were found in F_1_ plants of the crosses). The *L. japonicus* ecotype Gifu B-129 was grown at 20–22 °C with a 16-h light/8-h dark photoperiod at 150 μmol m^−2^ s^−1^ in the greenhouse.

### Scanning electron microscopy

Mungbean shoot apices at different developmental stages were fixed in FAA solution containing 3.7% (v/v) formaldehyde, 50% (v/v) ethanol, and 5% (v/v) acetic acid. Before vacuum freeze drying, fixed samples were dehydrated in an ethanol/tert-butanol series. The materials were transferred to a vacuum freeze dryer overnight. The preparation of shoot apices for scanning electron microscopy (SEM) analysis was as described by Chen et al.^24^. Samples were examined in a JEOL JSM-6360LV (JEOL) SEM at 10–15 kV of acceleration voltage.

### Molecular cloning of the full-length *LFY* gene (*VrLFY*) from mungbean

To clone the full-length mungbean *LFY* gene, we first searched the genome sequence database of mungbean (http://plantgenomics.snu.ac.kr) using sequences for *UNI* and *SGL1*. Finally, sequence alignment with *UNI/SGL1/LFY/FLO* coding sequences allowed the open reading frame of *VrLFY* to be defined. Polymerase chain reaction (PCR) was carried out using the primers in Supplementary Table 3. PCR products were cloned into the pGEM-T easy vector (Promega), and inserts were characterized by nucleotide sequencing.

### In situ hybridization

For in situ probes, gene-specific regions of *VrLFY*, *VrKNOXI*, and *LjKNOXI* genes were generated by PCR with primer sets (see Supplemental Table 4) and cloned into a pGEM-T vector (Promega, A1360). In situ probes were synthesized from these clones by in vitro transcription using the Digoxigenin RNA Labeling Kit (Roche) from either the T7 or SP6 promoter flanking the insert, generating either sense or anti-sense probes. Mungbean shoot apices were fixed overnight in 4 % (wt/vol) paraformaldehyde, pH 7.0, and then embedded in Paraplast (Sigma). RNase-free slices of the shoot apices were hybridized to digoxigenin-labeled probes and used for subsequent immunological detection as previously described^25^.

### Expression analysis by quantitative reverse transcription PCR

Shoot apices from 2-week-old mutant and wild-type plants were collected in RNase-free tubes and stored in a −80 °C freezer. Samples were taken in triplicate as biological replicates. Total RNA was extracted using the Plant RNA Kit (Omega) following the manufacturer’s instructions. Samples were then treated with RNase-free DNase I (Promega) for 30 min.

For quantitative reverse transcription PCR (qRT-PCR), first-strand cDNA was synthesized from total RNA using the First Strand cDNA Synthesis Kit (Fermentas). Real-time RT-PCR analysis was performed as three technical replicates in 384-well plates using SYBR® Premix ExTaq™ (Takara), on an ABI StepOnePlus machine, according to the manufacturer’s protocol (Applied Biosystems). The relative expression level of genes was determined by the 2^−ΔΔCT^ method. Amplification of *VrTUB* (*Vradi05g13910*), a constitutively expressed gene, was used as an internal control to normalize all data^26^. Shoot apices from a single genotype were represented by nine samples; independent total RNA isolations were generated from the three biological replicates, and three technical qRT-PCR replicates were performed on each of the total RNA preparations. The primers used for qRT-PCR are given in Supplementary Table 5.

### Transcript profiling by deep-sequencing

For Illumina sequencing, mRNA was purified from shoot apices of 2-week-old seedlings of *un1-1* mutant and wild-type plants and then fragmented into small pieces. Random hexamer primers and reverse transcriptase (Invitrogen) were used to carry out first-strand cDNA synthesis. Second-strand cDNA synthesis was performed with DNA polymerase I (New England BioLabs) after RNase H (Invitrogen) treatment. Four cDNA libraries were constructed, and cDNA sequencing was conducted using the Illumina HiSeq X Ten system according to the manufacturer’s protocol, with read lengths of 150 bp. The raw data were submitted to the NCBI Short Read Archive (SRP110723). The number of reads per kilobase of exon region in a gene per million mapped reads (RPKM) was used to normalize the gene expression data^27,28^. Differentially expressed genes were determined between the wild type and mutants according to statistical analysis of the frequency of each transcript, and their corresponding P-values were calculated^25^. The significance threshold of *P*-values in multiple tests was set by the false discovery rate (FDR). We used a FDR ≤ 0.05 and an absolute value of |log_2_ ratio| ≥ 1 as the threshold to judge the significance of gene expression differences.

### Phylogenetic analysis

The phylogenetic tree was constructed using the Molecular Evolutionary Genetics Analysis software (MEGA; version 6.0) by the neighbor-joining method (JFF Matrix model) with 1000 bootstrap replications.

### Data availability

Sequence data from this article can be found in the GenBank data libraries under the following accession numbers: XP_014491863.1 (VrLFY); XP_017441945.1 (VaLFY); XP_007137848.1 (PhvLFY); XP_003526918.2 (GmLFY1); XP_014630701.1 (GmLFY2); AAX13294.1 (PFM); AAC49782.1 (UNI); XP_003602745.1 (SGL1); XP_002284664.1 (VFL); AF197934_1 (FALSIFLORA); AAM27941.1 (LEAFY); AAA62574.1 (FLORICAULA); AQQ16908.1 (ChLFY); XP_015635355.1 (RFL); and O04407.1 (NEEDLY).

## Results

### Compound leaf development in mungbean

**Fig. 1:**
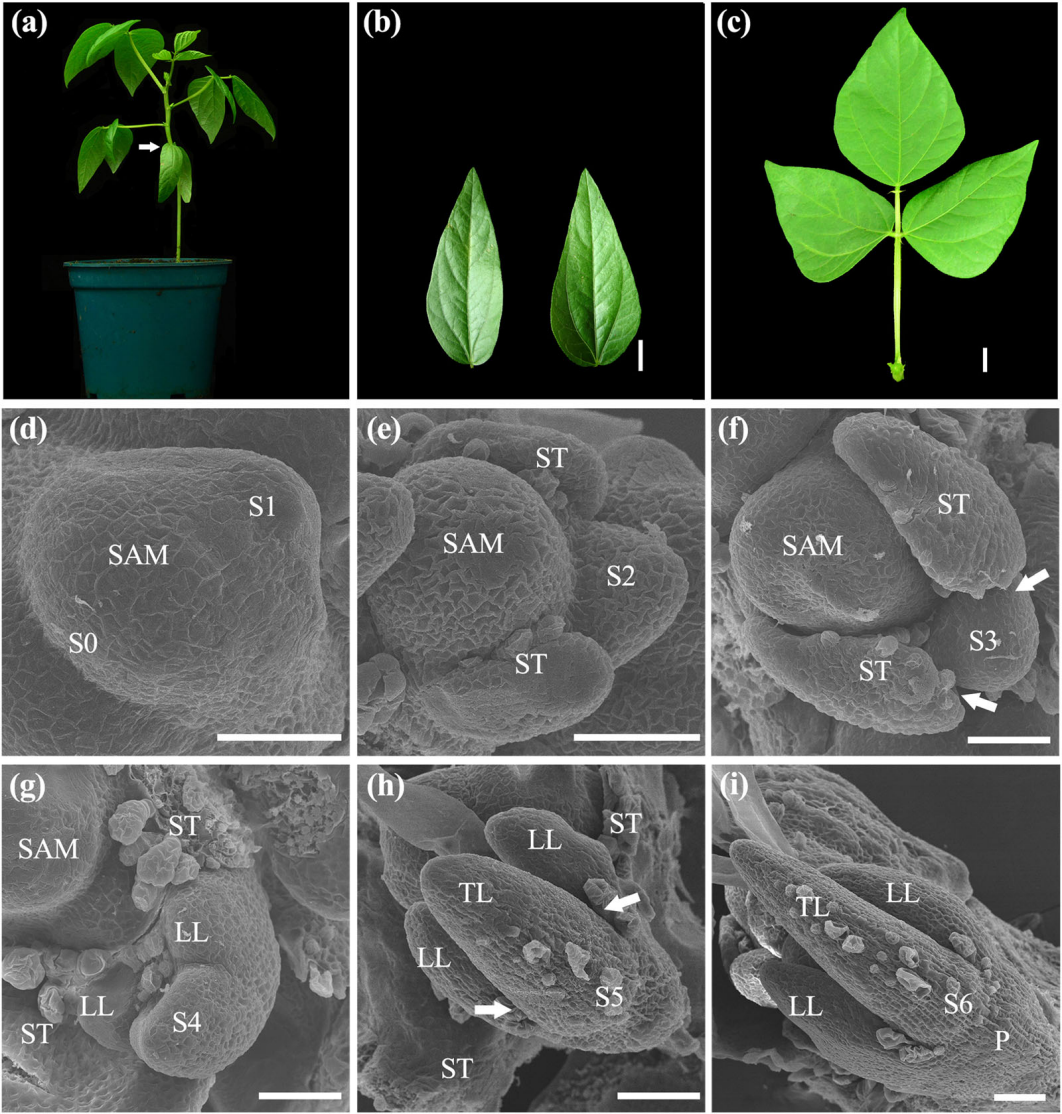
The ontogeny of compound leaf development in wild-type mungbean. **a** Whole plant morphology of mungbean. The arrow indicates the opposite juvenile leaves at the first node. **b** A pair of juvenile leaves of mungbean. **c** Adult compound leaf of mungbean. **d**–**i** SEM analysis of compound leaf development. **d** Sites of the incipient leaf primordia were specified at the periphery of the SAM at S0. At S1, a common leaf primordium was initiated as a strip of cells outgrowing along the periphery of SAM. **e** A pair of stipule primordia (ST) was initiated from the proximal end of the common leaf primordium at S2. **f** At S3, the boundaries (arrows) between the stipule and lateral leaflet primordia were formed. **g** At S4, a pair of lateral leaflet primordia (LL) emerged between the stipule and common leaf primordium. **h** At S5, the common leaf primordium differentiated into a terminal leaflet primordium (TL) as indicated by development of trichomes from the abaxial surface. Boundaries (arrows) were formed between the lateral and terminal leaflet primordia. **i** At S6, the leaflet primordia folded as a result of outgrowth of the abaxial surface, and the region between the stipule and lateral leaflet primordia expanded to form a petiole (P). Trichomes developed from the abaxial surface of both the stipule and lateral leaflet primordia. **b**, **c** Scale bars = 1 cm; **d**–**i** Scale bars = 50 μm

Similar to *M. truncatula*, *L. japonicus*, and other compound leaf-producing species, leaf development in mungbean was heteroblastic. The first pair of juvenile leaves with simple leaf morphologies emerged in opposite phyllotaxy on the first node of a developing mungbean plant and was succeeded by adult trifoliate leaves in alternate phyllotaxy (Fig. 1a–c). The wild-type trifoliate leaves of mungbean consisted of a pair of stipules, a petiole, 2 lateral leaflets, a rachis, and a single terminal leaflet (Fig. 1c). To facilitate the characterization of leaf mutants in mungbean, we investigated leaf developmental processes by scanning electron microscopy (SEM). The morphological changes during compound leaf development in mungbean can be divided into seven distinct stages. At Stage 0 (S0), cells along the periphery of SAM were recruited as founder cells and became an incipient leaf primordium (Fig. 1d). At S1, a common leaf primordium formed as a strip of cells grew out along the periphery of SAM (Fig. 1d). At the subsequent S2, a pair of stipule primordia emerged at the proximal end of the common leaf primordium (Fig. 1e), enlarged, and then separated away from the common leaf primordium so that boundaries were established between the two stipules and the common leaf primordium at S3 (Fig. 1f). At S4, a pair of lateral leaflet primordia emerged between the stipule and the common leaf primordium; and then the common leaf primordium differentiated into a terminal leaflet primordium (Fig. 1g). Subsequently, at S5, the lateral leaflet primordia were separated away from a terminal leaflet primordium so that a boundary was established, and trichomes initiated from the abaxial surface of the terminal leaflet primordium (Fig. 1h). Following S5, the lateral leaflets and terminal leaflet primordium became folded (Fig. 1i), and the region between the stipule and lateral leaflet primordia expanded to become a petiole as a result of cell division and cell expansion at the S6 stage.

### Isolation and characterization of *un* mutants in mungbean

**Fig. 2:**
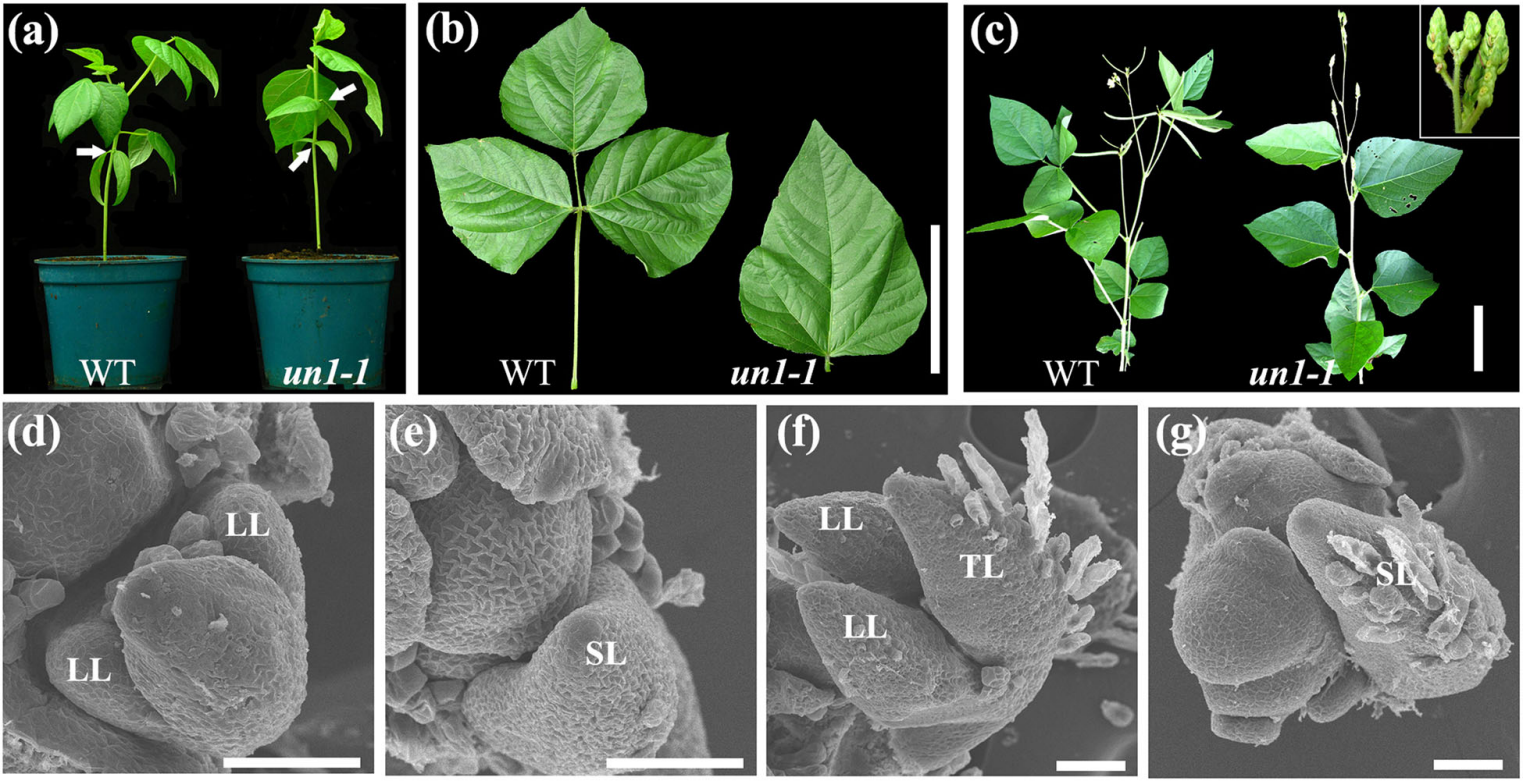
Phenotypes of mungbean *unifoliate* (un) mutants. **a** Wild-type mungbean (left) and *un1-1* mutant (right) exhibiting compound and simple leaf forms, respectively. Arrows indicate the opposite juvenile leaves at the first node in the wild type and at the first and second nodes in the *un1-1* mutant. **b** Close-up views of the adult leaves of the wild type (left) and *un1-1* mutant (right). **c** Morphology of a mature *un1-1* mutant (right), exhibiting simple leaf and floral homeotic phenotypes compared to the wild type (left). The inset has a close-up view of the inflorescence of the *un1-1* mutant. **d**, **f** Leaf development of wild type at S4 and S6. **e**, **h** Leaf development of *un1-1* at S4 and S6. The lateral leaflet primordia did not form at the proximal end of the common leaf primordium. LL lateral leaflet, TL terminal leaflet, SL single leaflet. **b**–**c** Scale bars = 10 cm; **d**–**f** 50 μm indicated

Four similar leaf mutants (Supplementary Table 1), which mimicked the phenotype of the classical mutant *un*, were isolated from a mutant population of mungbean generated by gamma irradiation^29^. Genetic analyses demonstrated that they were allelic (Materials and Methods section). In these mutants, all adult leaves consisted of a short petiole bearing a single terminal leaflet (Fig. 2a, b). Compared with the terminal leaflet of the wild type, the single terminal leaflet of the mungbean *un* mutant was larger (Fig. 2b). We found that the heteroblastic progression was also delayed in *un* mutants such that two simple leaves were produced at the first and second nodes in opposite phyllotaxis. Flowers that developed in *un* mutants were abnormal and infertile. *un* mutants produced sepal-like proliferating structures that lacked petals and stamens, and the number of whorls of organs within the flowers was increased (Fig. 2c). Because of their infertility, *un* mutants were maintained as heterozygotes. Progeny from self-pollination of heterozygous lines segregated in a 3:1 ratio (33 wild-type plants and 9 mutant plants, *χ*^2^ = 0.28 < *χ*^2^_0.05_ = 5.99), suggesting that the mutant phenotype was controlled by a single-recessive gene.

To characterize leaf development defects in mungbean *un* mutants, SEM analysis of leaf development was conducted. This analysis indicated that in *un* mutants, leaf development was initially normal until S4, at which point, the pair of lateral leaflet primordia failed to initiate between the stipule and the common leaf primordium (Fig. 2d, e). All four alleles of the mungbean *un* mutants exhibited this identical defect. The defect in the initiation of the lateral leaflet primordia was persistent throughout subsequent developmental stages, resulting in the formation of simple adult leaves in the *un* mutants of mungbean (Fig. 2f, g).

### Molecular cloning of *VrLFY* in mungbean

**Fig. 3:**
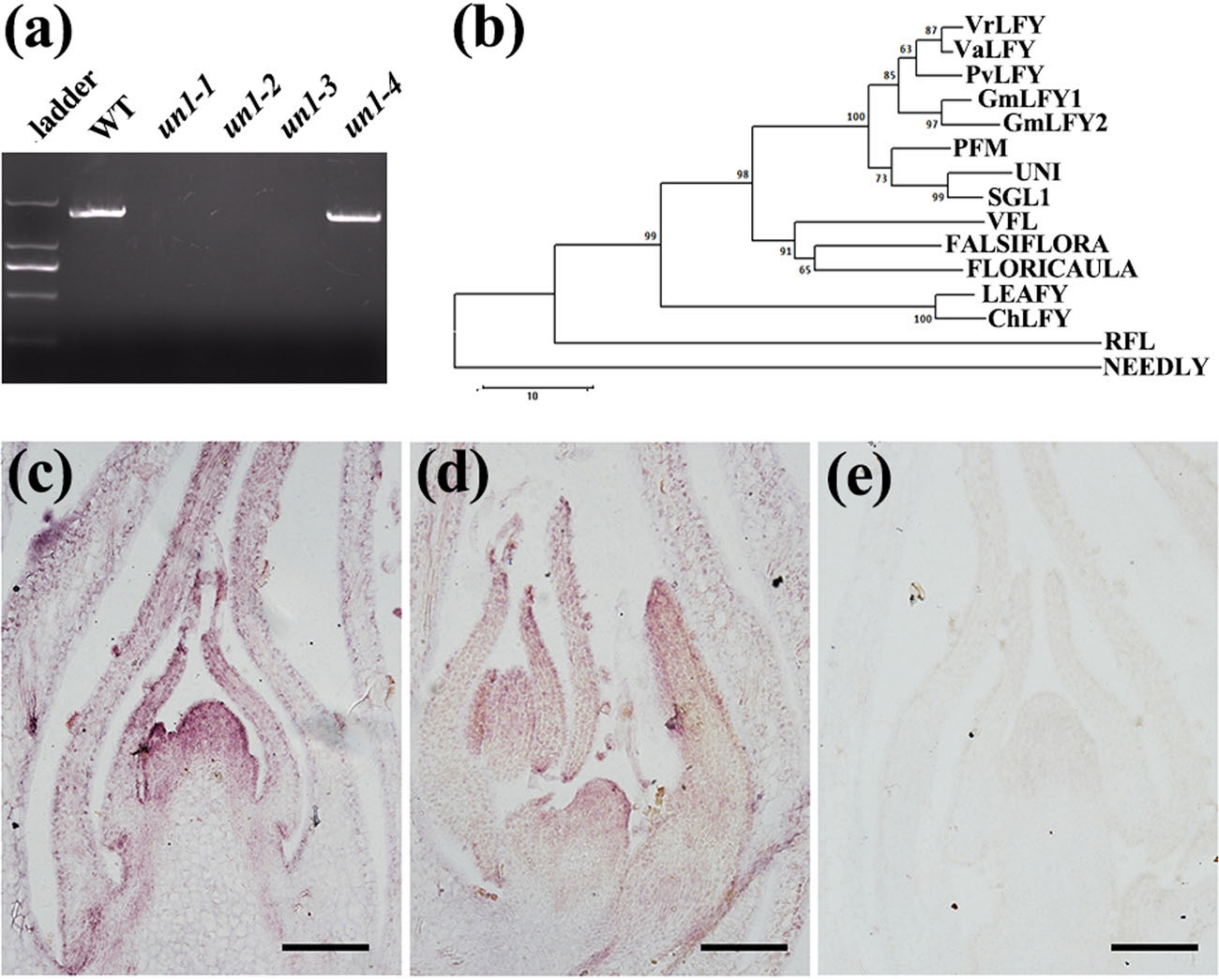
Molecular cloning of the VrLFY gene from mungbean. **a** PCR amplification of the *VrLFY* gene from wild-type mungbean and *un* mutants (WT, *un1-1*, *un1-2*, *un1-3*, and *un1-4*). Deletions were detected as no product on attempted amplification of the *VrLFY* gene from three mutant alleles. **b** Phylogenetic analysis of VrLFY and its putative orthologs: VaLFY of *V. angularis*, PvLFY of *Phaseolus vulgaris*, GmLFY1 and GmLFY2 of soybean, PFM of *L. japonicas*, UNI of pea, SGL1 of *M. truncatula*, VFL of *Vitis vinifera*, FALSIFLORA of tomato, FLORICAULA of snapdragon, LEAFY of *Arabidopsis*, ChLFY of *C. hirsute*, RFL of rice and NEEDLY of *Pinus radiata*. Bootstrap supports above 50 % from 1000 replicates are shown. **c**, **d** *VrLFY* gene expression was detected in SAM and developing leaf primordia. **e** The *VrLFY* sense probes were used as a negative control, and no hybridization signal was detected in SAM and leaf primordia. Scale bars = 100 μm

The floral homeotic defects and single-leaf phenotype of the *un* mutants resembled that of the *uni* mutant in pea and the *sgl1* mutant in *M. truncatula*^15,16^. The full-length DNA sequence of the *LFY* ortholog (*VrLFY*) in mungbean was obtained from the mungbean genomic database; the genomic sequence of *VrLFY* was 2155 bp in length. Alignment of the genomic sequence of *VrLFY* with its cDNA sequence showed the existence of two introns (Supplementary Figure 1a). PCR amplification of mungbean genomic *LFY* from the *un* mutants and from the wild-type plants indicated that three *un* alleles (*un1-1*, *un1-2*, and *un1-3*) carried deletions (Fig. 3a). Nucleotide sequencing showed that another allele, the *un1-4* mutant, had only a single base-pair substitution from the wild type gene (GAA to GGA, Supplementary Figure 1a). This resulted in an amino acid change (E112G, where the acidic amino acid Glu was replaced by a neutral amino acid Gly) in the N terminal domain of the protein (Supplementary Figure 1b).

Segregation analysis of an F_2_ population of the *un1-1* allele indicated that 50 out of a total of 208 individuals were homozygous for the deletion and exhibited both simple leaf and floral homeotic defects, suggesting that the deletion in the corresponding *VrLFY* gene cosegregated with the mutant phenotype. Thus, the locus of *un* in mungbean was allelic to *VrLFY*, which encoded a putative plant-specific transcription factor closely related to UNI in pea and SGL1 in *M. truncatula* (Fig. 3b). In situ RNA hybridization data revealed that the *VrLFY* gene was expressed in the SAM and in the emerging leaf primordia (Fig. 3c) and showed relatively high expression in the distal portion of leaf primordia (Fig. 3d).

### Characterization and expression analysis of *STM/BP*-like *KNOXI* genes in mungbean

To characterize the *KNOXI* genes in mungbean, we conducted a BLAST search of its genome (http://plantgenomics.snu.ac.kr), verifying candidate genes with the public transcriptome dataset^30^. Sixteen KNOX proteins were identified from mungbean and these were divided into three classes (class I, class II, and class M) based on phylogenetic analysis (Supplementary Figure 2)^30^. Nine proteins were classified as STM-like KNOXI proteins (Vradi07g26830, Vradi10g07810 and Vradi06g03570), BP-like KNOXI protein (Vradi06g14320) and KNAT2/6-like KNOXI proteins (Vradi08g11380, Vradi03g07470, Vradi05g04350, Vradi11g09640, and Vradi0322S00070). Five KNOXII proteins were classified as KNAT3/4/5-like proteins (Vradi05g1039, Vradi05g03240, and Vradi07g21010) and KNAT7-like proteins (Vradi11g02470 and Vradi07g13210). In addition, there were two members of class M KNOX proteins found in our mungbean sequence search (Vradi01g05360 and Vradi11g11780).

**Fig. 4:**
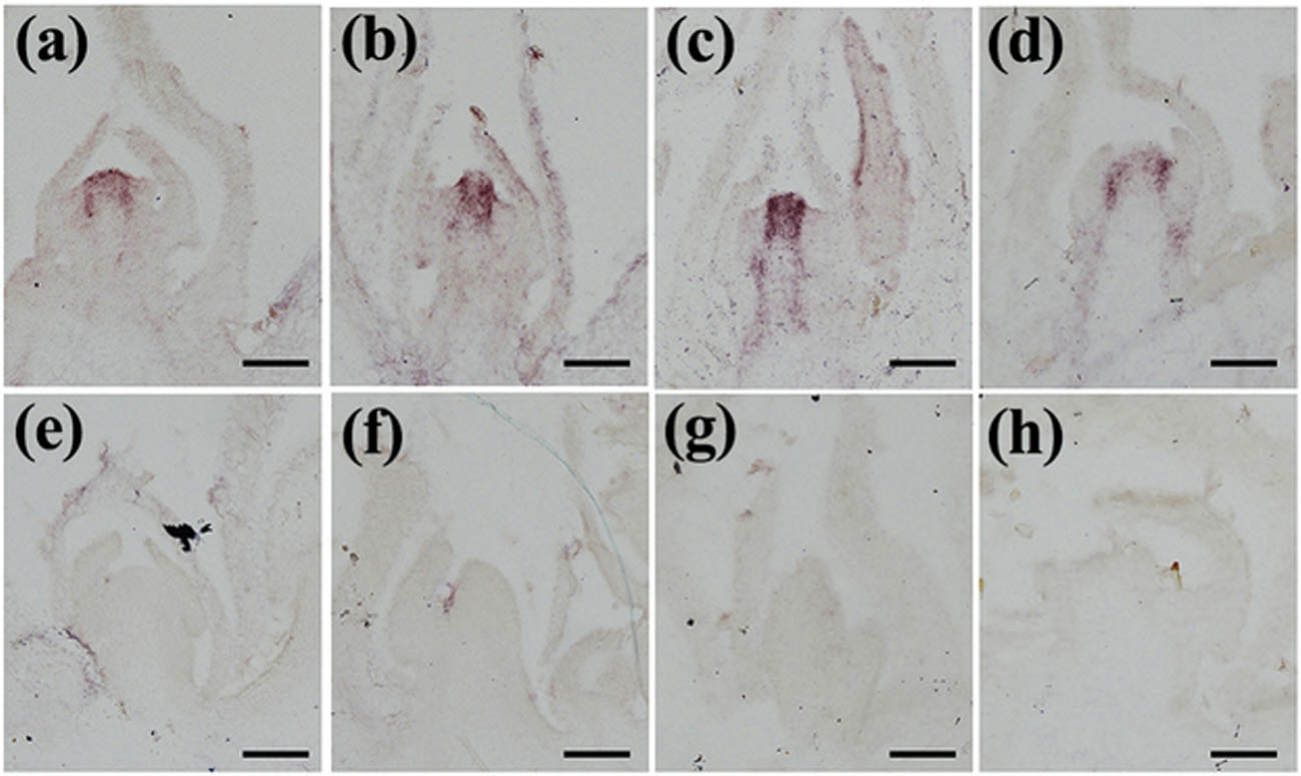
RNA in situ hybridization analysis of STM/BP-like *KNOXI* gene expression in mungbean. RNA in situ hybridization analysis of **a** *Vradi07g26830*, **b** *Vradi10g07810*, **c** *Vradi06g03570*, and **d** *Vradi06g14320* in the vegetative apices of mungbean. No expression was detected using the control sense probes of **e** *Vradi07g26830*, **f** *Vradi10g07810*, **g** *Vradi06g03570*, and **h** *Vradi06g14320*. Scale bars = 100 μm

It has been reported that *STM*/*BP*-like *KNOXI* genes in tomato and *C. hirsuta* are expressed in the compound leaf and are involved in the control of lateral leaflet development^4–6^. However, *STM/BP*-like *KNOXI* genes are not associated with compound leaf development in *M. truncatula* and pea^11–13^. Previously, accumulation of KNOXI proteins in compound leaf primordia of non-IRLC legumes was detected by polyclonal KNOXI-specific antibodies^12^. To compare the expression patterns of *STM/BP*-like *KNOXI* genes in mungbean with those of IRLC-legume and model plant species, in situ RNA hybridization of four *STM/BP*-like *KNOXI* genes (*Vradi07g26830*, *Vradi10g07810*, *Vradi06g03570*, and *Vradi06g14320*) was carried out on sections of apices from 2-week-old mungbean seedlings (Fig. 4). The results showed that there were different expression patterns among the four *STM/BP*-like genes in mungbean (Fig. 4a–d). The expression of the two *STM*-like genes *Vradi10g07810* and *Vradi06g03570* was strongly detected in the shoot apical meristem, and transcripts were also observed in the leaf primordia (Fig. 4b, c). However, expression of the *STM*-like gene *Vradi07g26830* and the *BP*-like gene *Vradi06g14320* was detected in the SAM but not in the leaf primordia (Fig. 4a, d).

Therefore, our data showed that the expression patterns of 2 *STM*-like *KNOXI* genes from mungbean differed from pea and *M. truncatula*, as in those species, no *STM*-like genes have been shown to be expressed in any stage of the compound leaf primordia^13^. Moreover, *BP*-like genes were not expressed in the compound leaf primordia of mungbean, *L. japonicus*, pea and *M. truncatula*, which is a different situation from the expression of these genes in *C. hirsuta* and tomato^4,5,7,11,13,22^.

### Transcript profiling of VrLFY downstream targets

To address the molecular function of *VrLFY* during leaf development, the transcriptome of shoot apices of 2-week-old seedlings from *un1-1* mutant and wild-type plants was studied using RNA-Seq. A total of 538 differentially expressed genes (300 downregulated and 238 upregulated) were identified between the mutant and wild-type plants (Supplementary Table 2). The results revealed a significant representation of genes associated with circadian rhythm and plant hormone signal transduction (Supplementary Table 2). In the *un1* mutants, the genes encoding proteins with high similarity to GIGANTEA 3 (GI3), GIGANTEA-like, Phytochrome A (PHYA), TIME OF CAB EXPRESSION 1 (TOC1), CIRCADIAN CLOCK ASSOCIATED 1 (CCA1), EARLY FLOWERING 3 (ELF3), and Adagio protein 3 (ADO3) were significantly downregulated, while those with high similarity to LATE ELONGATED HYPOCOTYL (LHY) were significantly upregulated. It has been reported that these are key factors in the regulation of the circadian clock and regulate important developmental transitions such as flowering, which was consistent with the involvement of LFY and its orthologs in controlling flowering time and phase transition^31–33^.

Previous studies have reported that plant hormones, including auxin and gibberellins (GA), play critical roles in leaflet initiation and compound leaf development^34,35^. In our expression studies, auxin, GA, ethylene and cytokinin-related genes were significantly differentially expressed in mutants compared to wild-type plants (Supplementary Table 2). In addition, a number of receptor-like protein kinases were differentially expressed, which might imply that several signaling cascades involved in cell proliferation and differentiation play a significant role in the control of compound leaf development in mungbean.

The transcripts of the *KNOX* family genes showed no obvious differential expression (data not shown), with the exception that one of the class M *KNOX* genes, *Vradi11g11780*, was decreased by approximately eightfold in vegetative shoot apices of the *un1-1* mutant compared with the wild-type plant (Supplementary Table 2), and this was confirmed by qRT-PCR (data not shown). It has been reported that increasing the expression of class M *KNOX* genes in *Arabidopsis* and tomato results in serrated leaves and a larger number of leaflets, respectively^36,37^.

### Genetic interactions affecting compound leaf development in mungbean mutants

**Fig. 5:**
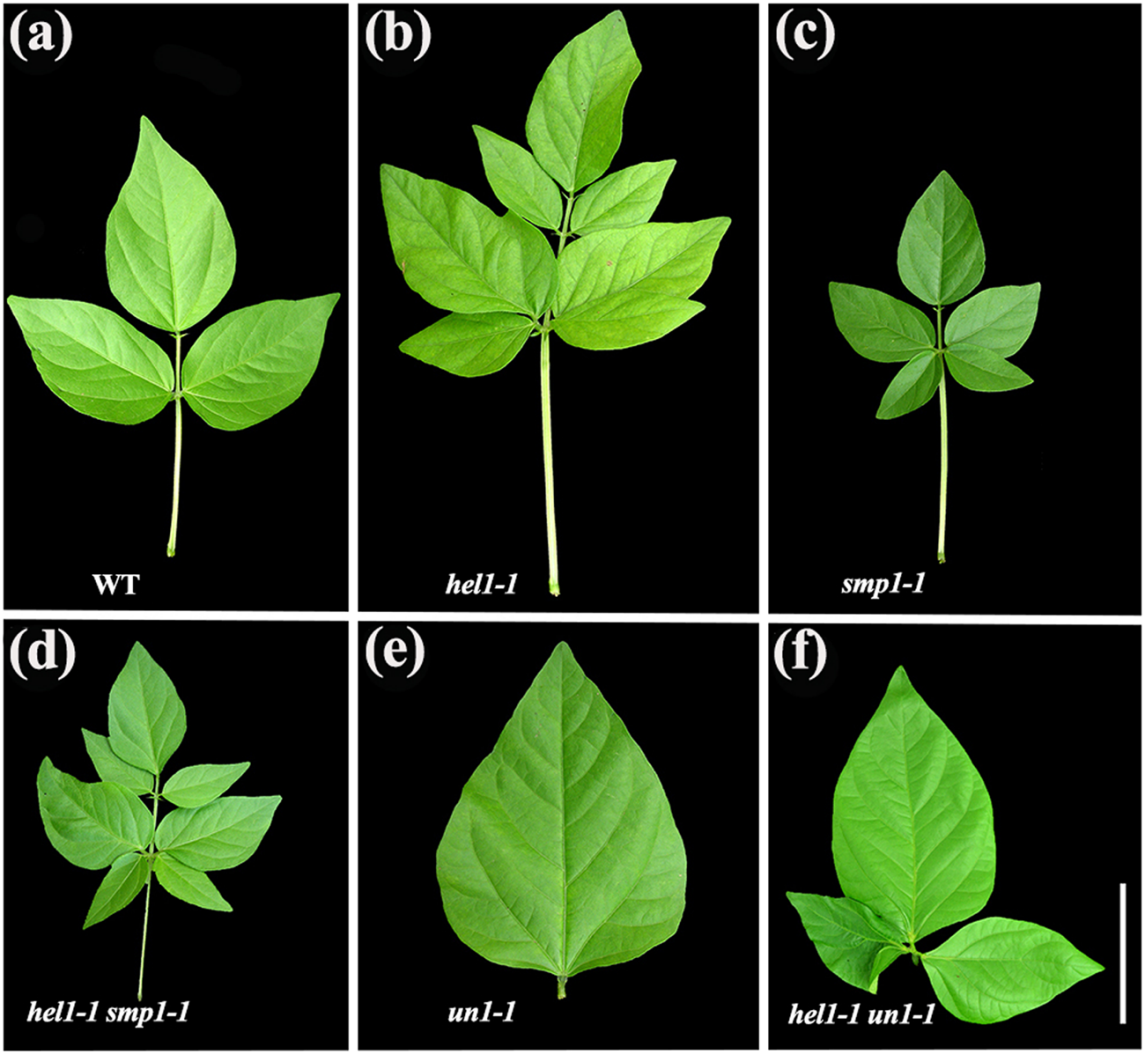
Genetic interactions among leaf mutants in mungbean. Compound leaf phenotype of the wild type and mutants. **a**–**f** Mature compound leaves of **a** WT, **b** *hel1-1*, **c** *smp1-1*, **d** *hel1-1 smp1-1*, **e** *un1-1*, and **f** *hel1-1 un1-1* mutants (all in the Sulu ecotype). Leaves of the *hel1-1 un1-1* double mutants exhibited three leaflets with short petioles. The *hel1-1 smp1-1* double mutants were heptafoliate leaves of small size, indicating an epistatic interaction between *hel1* and *smp1* in the control of leaflet number. Scale bars = 10 cm

Other mutants that showed an increase in the number of leaflets were identified, including *heptafoliate leaflets1* (*hel1*) and *small-pentafoliate leaflets1* (*smp1*), with 2 and 3 alleles, respectively (Fig. 5a–c; Supplementary Table 1). The juvenile leaves of the *hel1* mutant showed an extreme dissection and leaflet-like structures, sometimes associated with stipules, which developed in the proximal part of the blade (Supplementary Figure 3). The juvenile leaves of the *smp1* mutant were normal, but the adult leaves exhibited five leaflets of small size (Fig. 5c). We made crosses between *hel1* and *smp1* to examine genetic interactions. In the resulting F_2_ population, there were four classes of leaflet size and number: trifoliate, heptafoliate, small-heptafoliate, and small-pentafoliate. The numbers of plants in the different leaflet classes approximated a 9:3:3:1 ratio of the respective phenotypes (data not shown), suggesting that there were 2 unlinked genes controlling the multiple leaflet trait in mungbean. Further genetic analysis showed that the small-heptafoliate plants were *hel1 smp1* double mutants (Fig. 5d), indicating that there was no additive phenotype of the double mutation in terms of leaflet number and that *HEL1* interacted genetically with *SMP1* in the control of leaflet number in mungbean compound leaf development.

To genetically test the involvement of *un* in the proliferation of lateral leaflet primordia in the *hel1* mutant, we generated *hel1-1 un1-1* double mutants. Juvenile plants of the double mutant were similar to that of a *hel1* single mutant. However, all adult leaves in the *hel1-1 un1-1* double mutants consisted of a short petiole bearing two lateral leaflets and one terminal leaflet (Fig. 5f), indicating the requirement for the *VrLFY* gene in the proliferation of lateral leaflet primordia in the *hel1* mutant. In addition to the alterations in leaflet number, the *hel1* and *un* mutants also exhibited alterations in the proximal-distal axis of compound leaves. Compared with wild-type leaves, the petiole length of mature leaves in the *hel1* mutant was increased by approximately 10%, while the mature leaves of the *un* mutant exhibited short petioles (Fig. 5b, e). Interestingly, the petiole length of mature leaves of *hel1-1 un1-1* double mutants was short (Fig. 5f), resembling those of the *un* single mutant, suggesting that *un* is genetically epistatic to *hel1* in leaf petiole development.

### HEL1 regulates the expression of *VrLFY* and *STM/BP*-like *KNOXI* genes

**Fig. 6:**
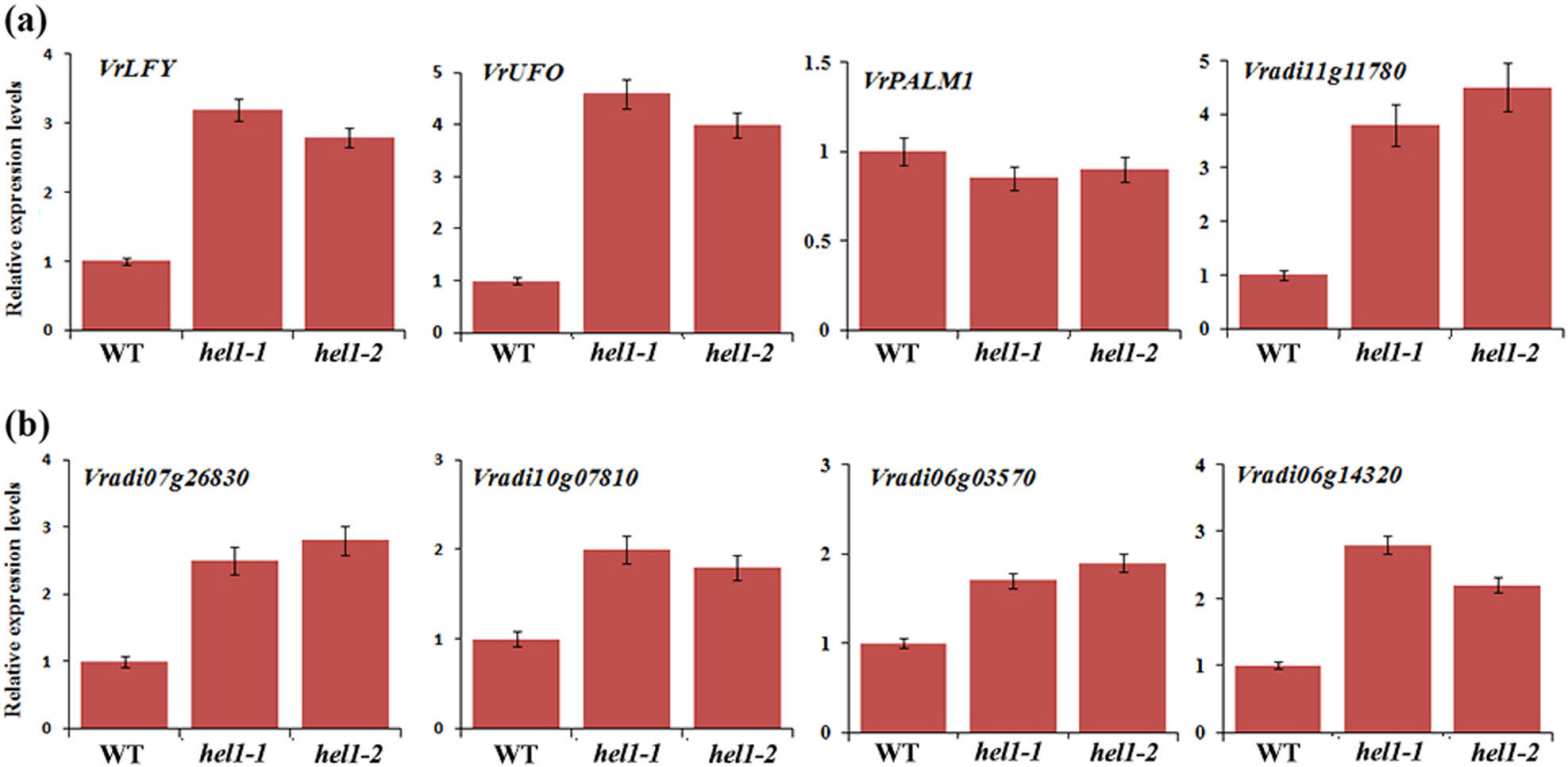
qRT-PCR analysis of key genes expressed in *hel* mutants. qRT-PCR analysis of gene expression relative to that of the mungbean *TUB* gene. The level of transcripts was examined in the shoot apices of mutants compared with wild-type plants 2 weeks after seed germination. Bars represent means ± SEs (*n* = 3). *Vradi07g26830* is a KNOXM gene. *Vradi07g26830*, *Vradi10g07810*, *Vradi06g03570*, and *Vradi06g14320* are *STM/BP*-like *KNOXI* genes

The genetic analysis described above indicated that *HEL1* controls trifoliate development via 2 distinct pathways, either dependent or independent of LFY. We first examined the expression of *VrLFY* and its putative upstream transcription factor *PALM1* (*VrPALM1*) and cofactor *UFO* (*VrUFO*) in the wild type and *hel1* mutants^17,18^. qRT-PCR data revealed that in vegetative shoot apices of the *hel1* mutant compared with wild-type plants, *VrLFY* and *VrUFO* transcript levels were increased by threefold and fourfold, respectively (Fig. 6a). However, *VrPALM1* transcript levels showed no obvious change in the *hel1* mutant compared with wild-type plants. Because some *KNOXI* genes expressed in the compound leaf were likely associated with compound leaf development in mungbean, we further examined the expression of *KNOX* genes in the *hel1* mutant and wild-type plants. The results showed that the expression of the 4 *STM/BP*-like *KNOXI* genes was significantly upregulated (Fig. 6b), while other *KNOXI* genes were not upregulated (data not shown). Interestingly, a *KNOXM* gene, *Vradi11g11780* (Supplementary Figure 2), was also upregulated by fourfold in the *hel1* mutant (Fig. 6a). Taken together, these data suggest that HEL1 regulates the expression of *LFY* and *KNOXI* genes to determine the leaflet number in mungbean.

## Discussion

### The role of the *LFY* orthologs in compound leaf development in legumes

In most compound-leafed species, activation of *KNOXI* gene expression in the leaf primordia is correlated with the development of compound leaves^6,12^. However, in IRLC legume species with compound leaves such as pea and *M. truncatula*, the *STM/BP*-like *KNOXI* genes are excluded from the leaf primordia, and their expression is not correlated with compound leaf development^11–13^. In these plants, the *LFY* orthologs appear to function in place of the *KNOXI* genes in controlling compound leaf development^12,15,16^. The pea *uni* mutants and *M. truncatula sgl1* mutants exhibit compound leaf defects, with all adult leaves changed to simple leaves, indicating that the *LFY* orthologs play a significant role in compound leaf development in IRLC legumes^15,16^.

The available information on the role of *LFY* orthologs in compound leaf development in non-IRLC legumes comes from phenotypic analysis of *L. japonicus pfm* mutants and soybean *LFY* transgenic lines^12,22^. In the *pfm* mutants, compound leaves lack 1 or 2 basal leaflets^22^. In transgenic soybean lines in which the endogenous *LFY* genes are downregulated, only the leaflet number of the compound leaves produced at the second node is reduced^12^. In tomato, the mutant of the *LFY* ortholog, *fa*, has a reduced number of small leaflets present on the compound leaf^32^. Recently, it has been reported that the *lfy* mutant in *C. hirsuta* shows a lower number of leaflets than the wild type^33^. Therefore, it seems that the single leaflet phenotype caused by mutations of the *LFY* orthologs is only exhibited in IRLC legumes. It is thought that the *LFY* orthologs acquired a more significant role no earlier than the divergence of the Hologalegina clade from the other legumes^12^.

However, our results showed that the *un* mutants in mungbean, with a complete conversion of compound leaves into simple leaves, were loss-of-function mutations of *VrLFY*. This finding indicated that *VrLFY* could play a significant rather than a minor role in compound leaf development in mungbean, a member of the non-IRLC legumes (Fig. 2a–c). Interestingly, mutants exhibiting simple leaves and malformed flowers have also been reported in other non-IRLC legumes, including adzuki bean and cowpea^38,39^. While unproven, one could speculate that some of these mutant phenotypes in adzuki bean and cowpea, with similarities to the *un* mutants in mungbean, could also be caused by mutations of *LFY* orthologs. If so, *LFY* orthologs could play a significant role in compound leaf development in other non-IRLC legumes, not just in mungbean. In addition, the question of when in evolution *LFY* orthologs acquired a significant role in compound leaf development might need to be re-addressed^12^. Further investigation of the function of *LFY* orthologs in leaf development of different clades of non-IRLC legumes would be helpful in answering this question.

### Expression pattern of *STM/BP*-like *KNOXI* genes in legumes

Phylogenetic analysis showed that there were duplications of *KNOXI* genes in legumes (Supplementary Figure 2). There was 1 *STM* gene and 1 *BP* gene in *Arabidopsis*, tomato and *C. hirsuta*. However, in IRLC legumes such as *M. truncatula* and pea, there were 2 *STM*-like genes and 1 *BP*-like *KNOXI* gene. In non-IRLC legumes such as mungbean and *L. japonicus*, there were 3 *STM*-like genes and 1 *BP*-like *KNOXI* gene. *In situ* RNA hybridization showed that the 2 *STM*-like *KNOXI* genes in mungbean were expressed at the compound leaf primordia (Fig. 4), which was different from that of pea and *M. truncatula* in which none of the *STM*-like genes were expressed in any stage of the compound leaf primordia^11,13^. *BP*-like genes were not expressed in the compound leaf primordia of mungbean (Fig. 4) and *L. japonicus* or in pea and *M. truncatula*^5,7,9–11,13^. This result was in contrast to findings of *BP* orthologs from *C. hirsuta* and tomato, which were expressed in the compound leaf primordia^25^. It has been shown that differences in expression patterns between *BP* from *Arabidopsis* and *ChBP* from *C. hirsuta* are attributable to their cis-regulatory regions^7^. When *KNOXI* genes are overexpressed in *M. truncatula* and *Alfalfa*, there is an increase in leaflet number^12,13^. The loss of the role for *KNOXI* genes in compound leaf development of IRLC legumes also likely occurred due to a loss of expression^12,13^. However, this loss of expression of the *STM*-like and *BP*-like genes in compound leaf primordia in IRLC legumes could have occurred at different times in evolution because *BP*-like genes were also not expressed in compound leaf primordia in non-IRLC legumes, such as mungbean and *L. japonicus*.

### VrLFY could interact with KNOXI in mungbean to regulate compound leaf development

In simple-leafed species, *LFY* orthologs play a key role in phase transition and floral development. Many downstream targets and DNA binding motifs of LFY have been identified at the genomic level in the model plant *Arabidopsis*^40,41^. Despite its key role in compound leaf development in species such as pea and *M. truncatula*, how the LFY orthologs regulate downstream genes to affect lateral organ development, especially that of compound leaf development, remains elusive. In this study, transcriptomic analysis uncovered a total of 538 differentially expressed genes between mutants and the wild type. Several types of key factors, such as *CCA1* and *ELF3*, involved in flowering time and phase transition were among the significantly differentially expressed genes (Supplementary Table 2), which was consistent with the conserved function of LFY orthologs in plants.

LFY has been reported to regulate the expression of some *KNOXI* genes such as *BP* and *KNAT2* in *Arabidopsis* during pedicel and flower development^41,42^. However, the transcription levels of the *KNOXI* genes showed no obvious change in the *un* mutant compared with the wild-type plant (data not shown), suggesting that *VrLFY* does not regulate the expression of *KNOXI* genes at the transcriptional level in mungbean. Nevertheless, 1 class M *KNOX* gene, *Vradi11g11780*, with high similarity to *PETROSELIUM* (*PTS*) in tomato and *FUSED COMPOUND LEAF* (*FCL2*) in *M. truncatula*, was downregulated 3.5-fold in the *un1-1* mutant^37,43^. It has been shown that the class M KNOX proteins in *Arabidopsis* and tomato could form heterodimers with BEL1-like homeodomain (BELL) proteins and interfere with the regulatory networks of KNOXI-BELL complexes during leaf development^36^. Transgenic *Arabidopsis* lines overexpressing *KNATM-B* exhibit serrated leaves, and a mutant with upregulated expression of the *PTS* gene in tomato exhibits a proliferation of compound leaves^36,37^. In *M. truncatula*, the class M *KNOX* gene *FCL1* has been shown to control boundary establishment and petiole length of compound leaves and is required for the development of extra leaflet primordia in a *palm1* mutant^44^. Therefore, our results indicated that *VrLFY* might modulate KNOXI regulatory networks by regulating the expression of a class M *KNOX* gene (*Vradi11g11780*) to control compound leaf development. Mutant databases for model legume plants such as *M*. *truncatula* and *L*. *japonicus* and legume crops such as soybean are available^45–47^. It would be worth identifying mutants of *Vradi11g11780/FCL2* orthologs and dissecting their roles in compound leaf development in different legumes. Furthermore, it will also be necessary to identify mutations of the *STM/BP*-like *KNOXI* genes in mungbean and other non-IRLC legumes to investigate gene and protein interactions between *KNOXI* genes and the *LFY* orthologs during compound leaf development in non-IRLC plants.

### HEL1 could orchestrate *VrLFY* and *KNOXI* to control compound leaf development in mungbean

Our results suggested that there were 2 distinct regulatory processes mediated by the LFY ortholog and KNOXI proteins during compound leaf development in mungbean. It also raised the question of how the two processes were coordinated during compound leaf development in mungbean. The *HEL1* gene was a key locus of mungbean in the control of leaflet number, whose mutation resulted in dissected juvenile leaves and heptafoliate adult leaves (Fig. 5b and Supplementary Figure 3). Double mutant analysis showed that *hel1* genetically interacted with *un* and *smp1* to control the leaflet number of the compound leaves, indicating that lateral leaflet formation in the *hel1* mutant was dependent not only on *LFY* but also on other regulators in the control of compound leaf development. Consistently, gene expression analysis showed that the *STM/BP*-like *KNOXI* genes and a *KNOXM* gene, *VrLFY*, as well as its putative cofactor *VrUFO*, were significantly upregulated in the *hel1* mutant compared to the wild type (Fig. 6). Therefore, these results suggested that HEL1 could coordinate the regulatory processes mediated by VrLFY and KNOXI to control compound leaf development in mungbean.

Interestingly, *heptafoliate-leaf*-*like* mutants similar to the *hel1* mutant in mungbean were also identified and characterized in other legumes, including soybean and cowpea^48,49^. In soybean, the seven-leaflet character is a single recessive trait conditioned by the *lf2* locus^45^. Preliminary mapping results in mungbean revealed that the *HEL1* gene was located to a region of chromosome 11, which showed synteny with the *lf2* locus in soybean (data not shown)^50^. Future work to clone the *HEL1* gene and its ortholog in soybean and related legumes could provide new insights into the molecular mechanisms orchestrating the two regulatory processes mediated by the *LFY* ortholog and *KNOXI* genes during compound leaf development in mungbean and other legumes.

## Electronic supplementary material

Supplementary Figure 1. LFY gene structure and alignment of protein sequences

Supplementary Figure 2. Phylogenetic analysis of members of KNOX gene family in mungbean, L. japonicus, M. truncatula, pea, and Arabidopsis

Figure S3

Supplementary Table 1 The information of wild type and mutant lines

Supplementary Table 2 The list of the differentiated expressed genes in un1-1 mutant compared with wild type

Supplementary Table 3 The list of primers for PCR detection in un mutants

Supplementary Table 4 The list of primers for in in situ hybridization

Supplementary Table 5 The list of primers for qRT-PCR
