# Supplementary figures and images for: Genetic control of compound leaf development in the mungbean (Vigna radiata L.)
Source: Hortic Res. 2019 Feb 1;6:23. doi: 10.1038/s41438-018-0088-0 (PMC6355865; doi:10.1038/s41438-018-0088-0)

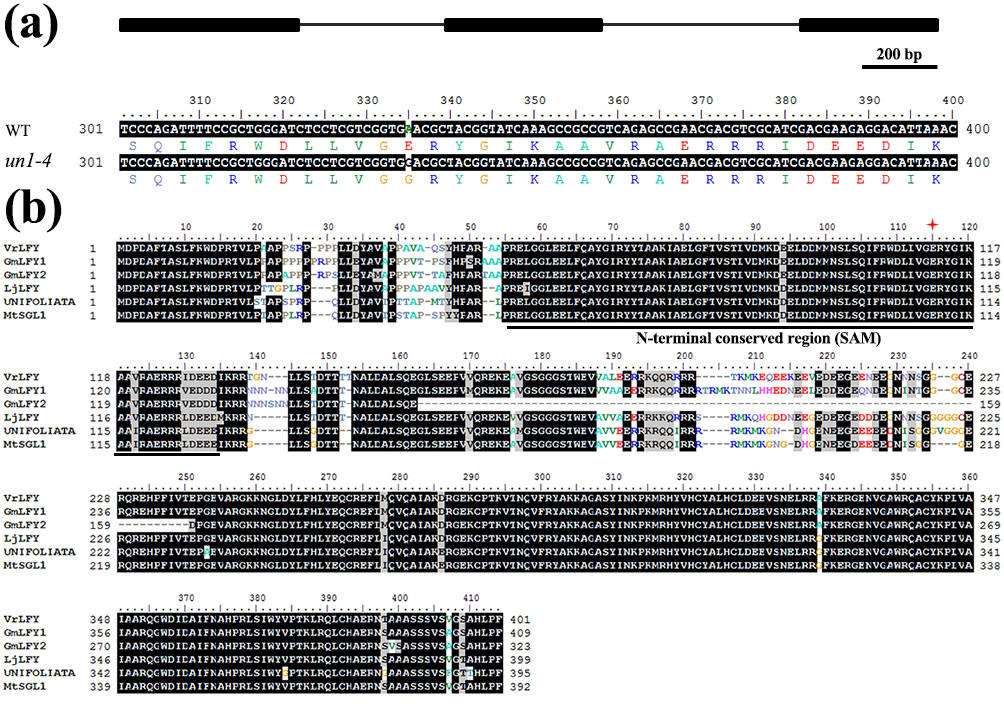

Supplement: Supplementary file 1 — Supplementary Figure 1. LFY gene structure and alignment of protein sequences [file 41438_2018_88_MOESM1_ESM.jpg]

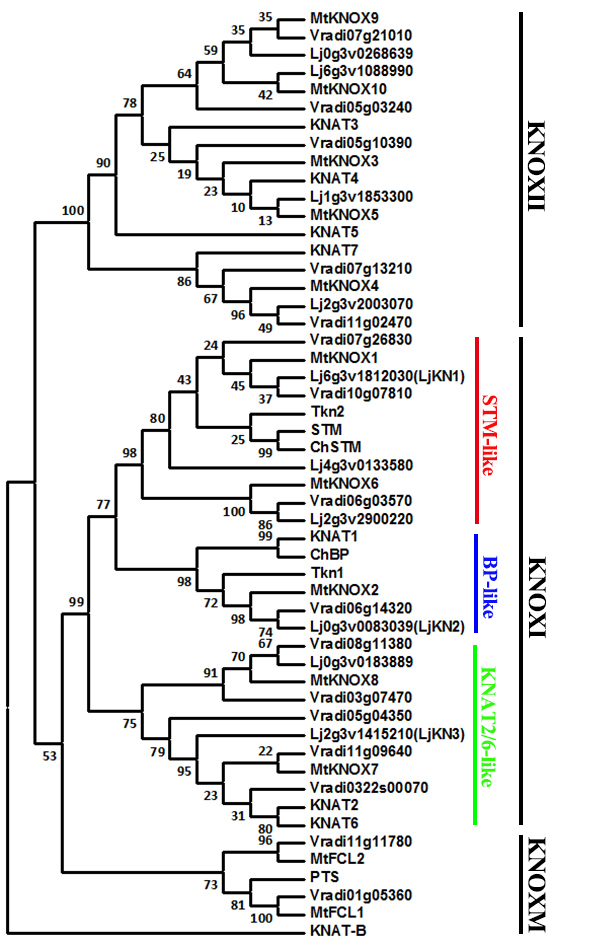

Supplement: Supplementary file 2 — Supplementary Figure 2. Phylogenetic analysis of members of KNOX gene family in mungbean, L. japonicus, M. truncatula, pea, and Arabidopsis [file 41438_2018_88_MOESM2_ESM.jpg]

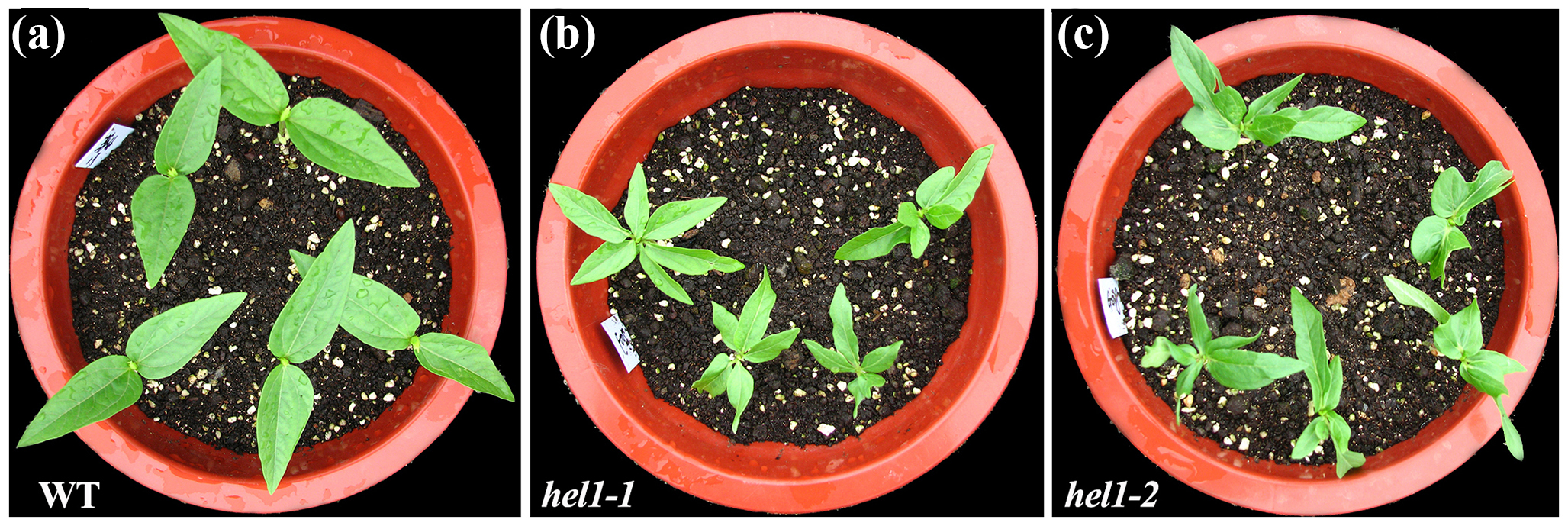

Supplement: Supplementary file 3 — Figure S3 [file 41438_2018_88_MOESM3_ESM.jpg]
